# Severe Pulmonary Artery Hypertension in Otherwise Silent Lupus: A Unique Hybrid Treatment Approach Using Hydroxychloroquine and Sildenafil

**DOI:** 10.7759/cureus.25411

**Published:** 2022-05-27

**Authors:** Maleeha Saleem, Sneha Kola, Rehan Shah

**Affiliations:** 1 Internal Medicine, Saint Francis Medical Center, Trenton, USA; 2 Internal Medicine - Rheumatology, Saint Francis Medical Center, Trenton, USA

**Keywords:** sle-associated pah, intrapulmonary shunting, hydroxychloroquine, cyclophosphamide, immunosuppression, right heart catheterization

## Abstract

Pulmonary hypertension (PH) is a rare manifestation of systemic lupus erythematosus (SLE). Even more rare is pulmonary artery hypertension (PAH) presenting as the initial manifestation of SLE and may be a cause of diagnostic delay. As symptoms of PAH are very mild in the early stages, prompt diagnosis is crucial to prevent the progression of the disease. Echocardiographic evaluation involving the measurement of different right-sided heart variables in addition to estimated pulmonary artery pressure helps in reducing the false-positive rates of detection of PAH. The role of immunosuppression in addition to PAH-specific vasodilator therapy is one of the key aspects of management to minimize flares and improve hemodynamics. Equally important is the choice of a regimen best suited to minimize complications. We present a case of PAH in newly diagnosed SLE and the diagnostic and treatment challenges as a safety net hospital.

## Introduction

Pulmonary hypertension (PH) is a complex disease with potentially lethal outcomes [1]. It is defined as an elevated mean pulmonary arterial pressure (mPAP) of 25 mmHg or greater at rest, and it has multiple underlying etiologies [1]. PH is classified into five categories according to the World Health Organization (WHO) Classification. The most common cause of PH is left heart disease (WHO group 2) [1]. Various connective tissue diseases (CTDs), including systemic sclerosis (SSc), systemic lupus erythematosus, rheumatoid arthritis (RA), or mixed connective tissue disease (MCTD), can also be associated with PH, and most of them fall in WHO group 1 [2]. In SLE, almost all of the five WHO classifications can be found [3]. Among the CTDs, SSc is considered the most common cause of pulmonary artery hypertension (PAH); however, SLE is also increasingly being recognized as associated with PAH [4]. As the symptoms of PAH in SLE can be mild and non-specific in the early stages, increasing awareness of this devastating complication is essential for early diagnosis [5]. The pathophysiology of PAH involves multiple mechanisms including vasoconstriction, vasculitis, in situ thrombosis, and endothelial dysfunction to interstitial lung disease leading to vasospasm [6]. Patients with unrecognized PAH or those who are not yet treated progress to right ventricular dilatation and failure, which can ultimately lead to death [6]. Here, we describe an interesting case of PAH in a patient with no established diagnosis of SLE and how our unique approach with hybrid therapy can be particularly helpful in severe SLE-associated PAH (SLE-a PAH) before referral to a tertiary care center.

The abstract of this case report was presented as a flat board poster at the American College of Cardiology conference in Washington in April 2022.

## Case presentation

A 60-year-old woman presented to the emergency room (ER) with progressively worsening exertional dyspnea for the past one week. She endorsed dyspnea even on positional changes like getting into and out of bed. She also endorsed generalized weakness, lower extremity swelling, orthopnea, and occasional paroxysmal nocturnal dyspnea; however, she denied any chest pain, shortness of breath at rest, wheezing, cough, fevers, palpitations, dizziness, syncope, weight gain, arthralgias, or myalgias. Her initial vitals showed oxygen saturation of 88% on room air with a blood pressure of 120/92 mmHg and a respiratory rate of 28 breaths per minute. On physical examination, there was jugular venous distention (JVD) along with a positive hepatojugular reflex and trace asymmetric pitting edema up to the mid shins. Her lungs were clear to auscultation, and on heart auscultation, there was an S3 gallop rhythm and a loud S2.

Her medical history was significant for unprovoked pulmonary embolism about two years ago for which she was treated with apixaban for six months. During that admission, rheumatologic testing showed positive anti-nuclear antibody (ANA) with a titer of 1:320, and the patient was asked to follow up with rheumatology; however, she was lost to follow up.

**Figure 1:**
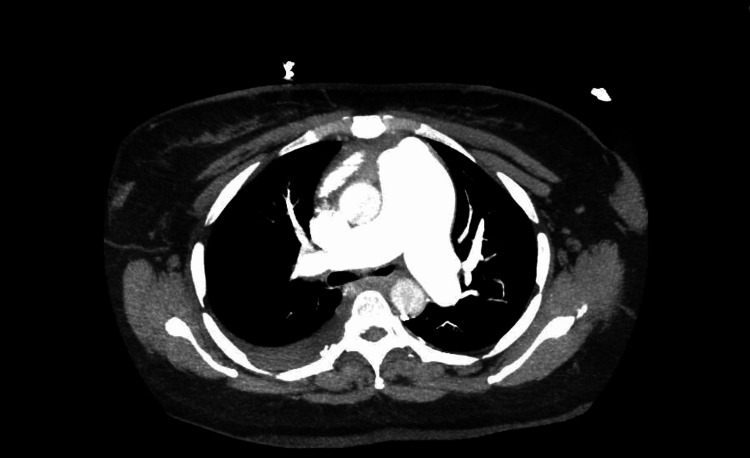
CTA of the chest showing no evidence of pulmonary embolism and enlarged diameter of pulmonary artery consistent with pulmonary hypertension. CTA: computed tomography angiography

Her lab workup showed high B-type natriuretic peptide of 948 pg/mL (normal range: <100 pg/mL), D-dimer was 4 pg/mL (normal: <0.50 pg/mL FEU) with an international normalized ratio of 1.3. Her erythrocyte sedimentation rate was 127 mm/hour (reference: 0-30 mm/hour) and C-reactive protein was 10 mg/dL (reference range: 0.02-1.00 mg/dL). An electrocardiogram obtained on admission showed P-wave >2.5 mm tall in II and >1.5 mm tall in V1 (P pulmonale) consistent with right atrial enlargement, right ventricular hypertrophy with RR-wave/S-wave ratio greater than 1 in lead V1, and deep T-wave inversions in V1 through V5 and right axis deviation. Computed tomography angiography of the chest with contrast showed no evidence of pulmonary embolism, PAH with right-sided heart enlargement, and right ventricular strain (Figure 1).

**Figure 2:**
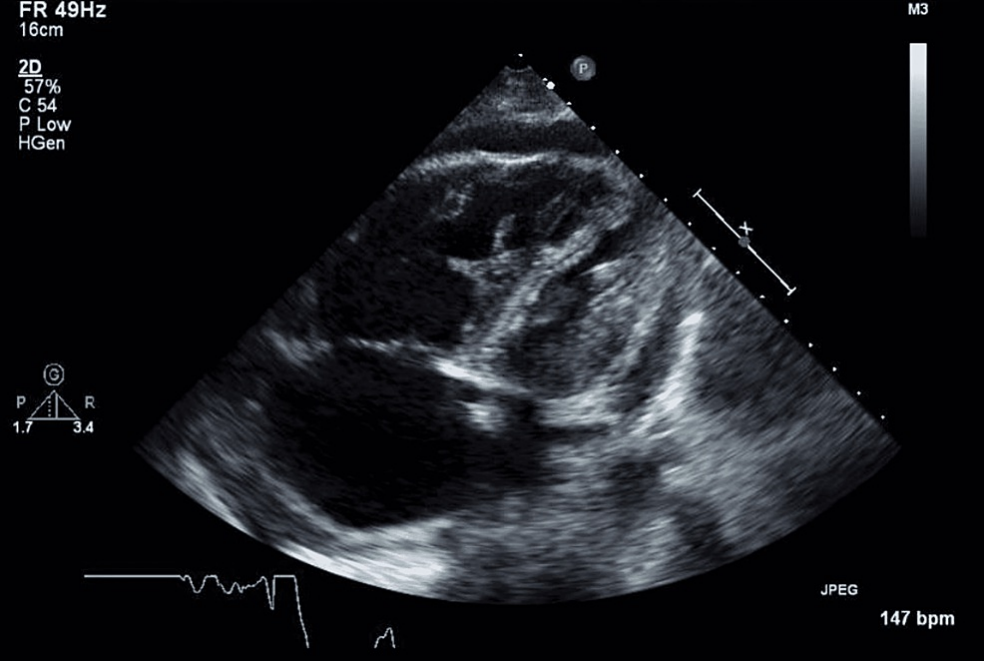
Dilated right ventricle with reduced global systolic function with elevated right ventricular end-diastolic pressure and abnormal motion of interventricular septum. Estimated: TRVmax 4.44 m/second, mPAP of 97 mmHg, and ePASP of 94 mmHg. TRVmax: tricuspid regurgitant velocity maximum; mPAP: mean pulmonary arterial pressure; ePASP: estimated pulmonary artery systolic pressure

The patient received multiple doses of furosemide on admission which was later held due to hypotension. Duplex ultrasound venous lower extremity showed right peroneal vein and right posterior tibial vein proximal to mid-segment subacute deep venous thrombosis (DVT). Transthoracic echocardiography (TTE) showed a normal left ventricular size and ejection fraction of about 60-65% with no wall motion abnormalities. The right ventricle was severely dilated, and global systolic function was severely reduced, as depicted in Figure 2. The septum had abnormal paradoxical motion consistent with right ventricular volume overload or elevated right ventricular end-diastolic pressure, tricuspid regurgitant velocity maximum (TRV max) of 4.44 m/second, mPAP of 97 mmHg, and estimated pulmonary artery systolic pressure of 94 mmHg. The diagnosis of severe PAH was confirmed using echocardiography and gold standard right heart catheterization (RHC) with pulmonary artery systolic pressure (PASP) of 94 mmHg, as shown in Table 1. Autoimmune workup showed positive ANA with a titer of 1:320 and homogenous staining pattern, as well as high anti-double-stranded (anti-DS DNA Ab) of 712 with normal complement levels. SLE was suspected based on four out of eleven American College of Rheumatology (ACR) criteria (pleuritis, lymphopenia, elevated ANA, and anti-DS-DNA Ab). The diagnosis of SLE was confirmed with an SLE disease activity index (SLEDAI) score of 8. Anti-centromere antibody, anti-phospholipid antibody, anti-cardiolipin antibody, lupus anticoagulant, angiotensin-converting enzyme (ACE), anti- ribonucleoprotein antibody (anti-RNP Ab), anti-Smith antibody, anti-SCL-70 antibody, HBsAg, anti-hepatitis C virus, and human immunodeficiency virus were all unremarkable.

**Table 1:** Hemodynamic parameters from Right heart catheterization: RAP = right atrial pressure, PAWP = pulmonary artery wedge pressure, PAP = pulmonary artery pressure, CO = cardiac output, CI = cardiac index, PVR = pulmonary vascular resistance RAP: right atrial pressure; PAWP: pulmonary arterial wedge pressure; PAP: pulmonary arterial pressure; CO: cardiac output; CI: cardiac index; PVR: pulmonary vascular resistance

Hemodynamic parameters	Reference range	Patient findings
RAP mmHg	2–6	12
PAWP mmHg	4–12	9
PAP mmHg	25/10	70/47
Mean PAP mmHg	25	59
CO L/minute	4–8	4.3
CI L/minute/m^2^	2.5–4	2.2
PVR woods unit	0.5–1.1	12.3

**Figure 3:**
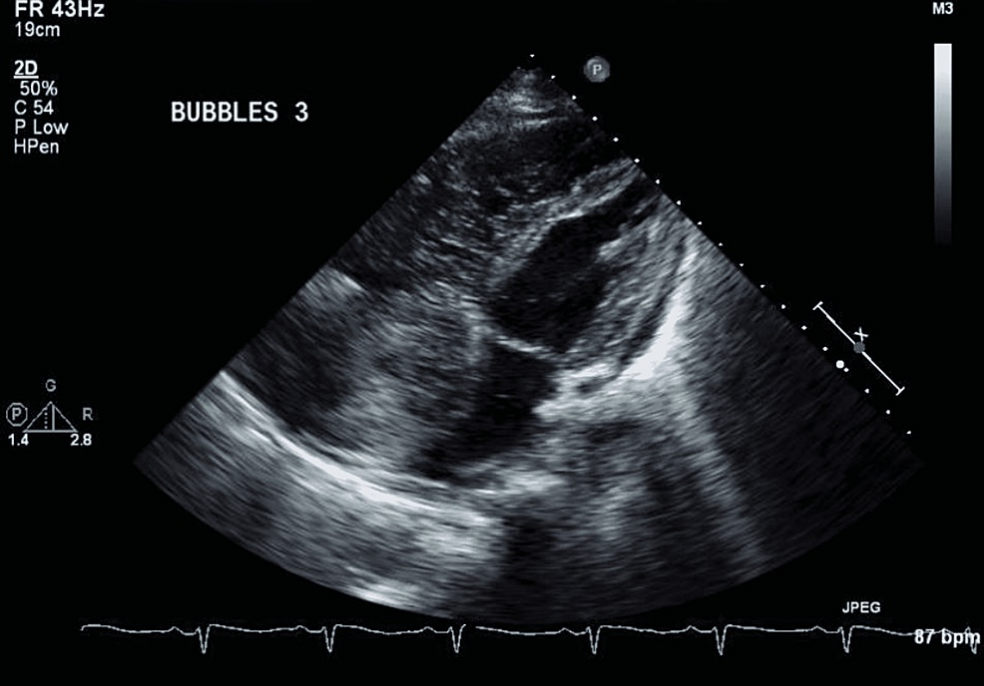
Bubble study demonstrating the late appearance of bubbles consistent with an intrapulmonary shunt.

Given these clinical and hemodynamic findings, she was diagnosed with PAH in the setting of SLE. She was started on hydroxychloroquine (HCQ) and sildenafil in consultation with pulmonology, cardiology, and rheumatology. The patient was also started on apixaban for DVT. An agitated saline study, as demonstrated in Figure 3, confirmed intrapulmonary shunting contributing to hypoxia, and the possibility of arteriovenous malformation was effectively ruled out as computed tomography with thin slices was negative. The patient’s ventilation-perfusion scan did not show any evidence of pulmonary thromboemboli.

Using shared decision-making, adjunctive treatment with a short course of steroids significantly improved the patient’s New York Heart Association (NYHA) functional class and hypoxia. We chose HCQ along with steroids for initial immunosuppression and sildenafil as initial vasodilator despite NYHA class IV due to better tolerability, relatively safe side effect profile, and limited experience with other PAH/SLE treatments in our community hospital. Our patient responded quite well to this combination, and her oxygen requirement reduced from high flow to 4-5 L on nasal cannula. The hybrid treatment approach effectively stabilized her for discharge on home oxygen of about 4 L. At the three-month follow-up at a PH tertiary care center, the addition of macitentan to the initial hybrid treatment further improved her hemodynamics, her NYHA functional class was reduced from 4 to 2, and her oxygen requirement was effectively reduced to room air. Repeat TTE during the six-month follow-up showed marked improvement of PASP from 97 mmHg to 67 mmHg.

## Discussion

PH without underlying parenchymal lung disease rarely occurs with symptomatic dyspnea or right-sided heart failure [7]. PH is a chronic disease defined as an increase in mPAP of ≥25 mmHg at rest and pulmonary vascular resistance of >3 Wood units [8]. According to the Registry to Evaluate Early and Long-Term Pulmonary Arterial Hypertension Disease Management (REVEAL), SLE has the second-highest prevalence of PAH after SSc [9]. As a non-invasive screening method, echocardiography is about 50% accurate in estimating right ventricular systolic pressure [10]. Considering epidemiology, the majority of patients with SLE-a PAH are women (95% in REVEAL with RHC confirmed disease) and a mean age of 45 years (45.5 ± 11.9) at PAH diagnosis [11]. Although PAH can be the first manifestation leading to SLE diagnosis, it is very rare [12]. The onset of PH in patients with SLE does not correlate with disease duration or the degree of extrapulmonary manifestations of the illness, and patients may present with PH in advance of their diagnosis of SLE [13]. In the late stages, right heart failure with liver enlargement due to congestion, ascites, and/or lower limb edema may be seen and this is when patients start becoming more symptomatic [13].

As the development of PAH in SLE carries a worse prognosis, prompt recognition and early treatment initiation are crucial [6]. TTE is recommended for the initial screening of patients with suspected PAH as well as for the evaluation of response to treatment [8]. The gold standard for diagnosis is RHC [8]. Vasoreactivity can be assessed with the use of nitric oxide, epoprostenol, or adenosine. Most patients with SLE are not vasoreactive, and therapy with calcium channel blockers has not proven to be useful [13]. According to the REVEAL risk score, our patient was at very high risk with one-year mortality of >30%.

Treatment of SLE-a PAH involves the use of immunosuppression as well as specific therapies including vasodilators [13]. Various parameters involving the severity of PAH on echocardiography, WHO classification functional class, exercise capacity, and hemodynamic and laboratory values are useful in tailoring individualized treatment approaches [13].

Intensive immunotherapy trials to evaluate treatment modalities and respective outcomes in SLE-a PAH used cyclophosphamide and steroids for immunosuppression [13]. Rituximab, mycophenolate mofetil, and cyclosporine have been used effectively in refractory cases of SLE-a PAH [12,14]. Similarly, among the vasodilators, for functional class III-IV with rapid progression of the disease and other markers of poor prognosis, initial therapy should be instituted with parenteral prostanoid agents, and then additional vasodilators are added as tolerated along with immunosuppression [6].

Our patient initially presented with exertional dyspnea and features of right-sided heart failure. TTE and RHC confirmed PAH. Her etiology of PAH was found to be SLE with an SLEDAI score of 8. Apart from vascular thrombosis and pleuritis, our patient did not have any other manifestations of SLE. This otherwise silent lupus is very rarely associated with severe PAH. She was started on HCQ and sildenafil in consultation with pulmonology, cardiology, and rheumatology. Using shared decision-making, adjunctive treatment with steroids and later on macitentan significantly improved NYHA functional class and hypoxia. SLE-a PAH is distinct compared with other CTDs associated with PAH in that it requires concomitant immunosuppression to prevent flares that can be life-threatening in a patient with severe PAH [15].

HCQ as immunosuppressive therapy and sildenafil as initial vasodilator therapy for SLE-a PAH has not been demonstrated in the literature. Patients should be referred to specialized centers early in the course of disease for PAH treatment and should be counseled in detail regarding the importance of compliance to therapy and regular follow-up. This case is of particular benefit for physicians, primarily in a community-based center, who might be dealing with such clinical scenarios in a limited setting. Moreover, our treatment approach might help them in selecting diverse immunosuppressive and vasodilator therapies.

## Conclusions

Treatment of SLE-a PAH using cyclophosphamide, rituximab, steroids, and prostanoids remains the mainstay of therapy. A modulated approach using HCQ along with sildenafil can be particularly useful in a subset of patients with SLE-a PAH with underlying intrapulmonary shunt and otherwise minimal disease activity to help provide hemodynamic (PAH) and disease (SLE) stability to limit morbidity before referring them to a tertiary care center.

## References

[1] Mensah KA, Yadav R, Trow TK, Brunet CM, Fares WH (2015). Lupus-associated pulmonary arterial hypertension: variable course and importance of prompt recognition. Case Rep Med.

[2] Strange G, Playford D, Stewart S, Deague JA, Nelson H, Kent A, Gabbay E (2012). Pulmonary hypertension: prevalence and mortality in the Armadale echocardiography cohort. Heart.

[3] Highland KB, Gilkeson G (2008). Pulmonary hypertension in systemic lupus erythematosus. Adv Pulm Hypertens.

[4] Wang J, Qian J, Wang Y (2017). Serological biomarkers as risk factors of SLE-associated pulmonary arterial hypertension: a systematic review and meta-analysis. Lupus.

[5] Prabu A, Gordon C (2013). Pulmonary arterial hypertension in SLE: what do we know?. Lupus.

[6] Kiani R, Siddiqui MD, Tantoush H (2020). Severe pulmonary hypertension as initial presentation of SLE: a case report and literature review. Case Rep Rheumatol.

[7] Prabu A, Patel K, Yee CS (2009). Prevalence and risk factors for pulmonary arterial hypertension in patients with lupus. Rheumatology (Oxford).

[8] Hoeper MM, Bogaard HJ, Condliffe R (2013). Definitions and diagnosis of pulmonary hypertension. J Am Coll Cardiol.

[9] Tselios K, Gladman DD, Urowitz MB (2017). Systemic lupus erythematosus and pulmonary arterial hypertension: links, risks, and management strategies. Open Access Rheumatol.

[10] Fisher MR, Forfia PR, Chamera E (2009). Accuracy of Doppler echocardiography in the hemodynamic assessment of pulmonary hypertension. Am J Respir Crit Care Med.

[11] Chung L, Liu J, Parsons L (2010). Characterization of connective tissue disease-associated pulmonary arterial hypertension from REVEAL: identifying systemic sclerosis as a unique phenotype. Chest.

[12] Prete M, Fatone MC, Vacca A, Racanelli V, Perosa F (2014). Severe pulmonary hypertension as the initial manifestation of systemic lupus erythematosus: a case report and review of the literature. Clin Exp Rheumatol.

[13] Dhala A (2012). Pulmonary arterial hypertension in systemic lupus erythematosus: current status and future direction. Clin Dev Immunol.

[14] Hennigan S, Channick RN, Silverman GJ (2008). Rituximab treatment of pulmonary arterial hypertension associated with systemic lupus erythematosus: a case report. Lupus.

[15] Jais X, Launay D, Yaici A (2008). Immunosuppressive therapy in lupus- and mixed connective tissue disease-associated pulmonary arterial hypertension: a retrospective analysis of twenty-three cases. Arthritis Rheum.

